# Editorial: Disruptive technologies for the study of host-pathogen interactions

**DOI:** 10.3389/fcimb.2022.1048342

**Published:** 2022-10-13

**Authors:** Juan F. Quintana, Clare Harding, Diego Huet, Adam Sateriale, Maria Bernabeu

**Affiliations:** ^1^ Wellcome Centre for Integrative Parasitology, University of Glasgow, Glasgow, United Kingdom; ^2^ Institute of Biodiversity, Animal Health and Comparative Medicine, University of Glasgow, Glasgow, United Kingdom; ^3^ Centre for Tropical and Emerging Global Diseases, University of Georgia, Athens, GA, United States; ^4^ Department of Pharmaceutical and Biomedical Sciences, University of Georgia, Athens, GA, United States; ^5^ The Francis Crick Institute, London, United Kingdom; ^6^ European Molecular Biology Laboratory (EMBL), Tissue Biology and Disease Modelling Unit, Barcelona, Spain

**Keywords:** host, pathogen, technologies, parasites, plasmodium, trypanosoma, *Cryptospordium* sp, gastrointestinal nematode (GIN)

## Introduction

Seminal discoveries in host-pathogen interactions have been catalysed by the development of new technologies. Microscopy could be considered the first disruptive technology in parasitology with the discovery of *Giardia*, for the first time in 1681 by Antoine Leeuwenhoek, the father of microscopy. It was not until two hundred years later that other parasitic protozoans were microscopically identified. Likewise, contemporary parasitology studies could not be envisioned without the establishment of *in vitro* culture conditions for parasites, mostly in the second half of the 20^th^ century, or the disruption of widely used molecular biology techniques, including PCR, sequencing or recombinant DNA technology or the development of computational and statistical approaches to analyse them. A new generation of cutting-edge, disruptive, and interdisciplinary technologies has emerged in the last decades, including protein-protein interactions at a nanoscale resolution, long read sequencing, whole cleared organ imaging at a mesoscale level, and stem-cell derived human and animal organoids. These have paved the way for basic discoveries in cell biology and have been applied to many research fields, from developmental biology to immunology. The adoption and implementation of such “disruptive technologies” has the potential to revolutionise the field of parasitology, transforming the way in which we explore and understand the intimate crosstalk established between parasites and their hosts during infection. It is likely that these disruptive technologies will render unprecedented insights into these otherwise devastating and often neglected diseases.

This Research Topic encompasses a series of original articles and reviews summarising key basic knowledge derived from the implementation of disruptive technologies to study a wide range of parasitic infections, from protozoan parasites such as *Cryptosporidium*, *Plasmodium*, and *Trypanosoma*, to gastrointestinal parasitic nematodes such as *Trichuris muris*. Mkandawire and Sateriale describe the application of a new generation of molecular biology approaches and discuss current challenges in *Cryptosporidium* research. Amongst those challenges, they point out the lack of genomic and transcriptomics data to understand virulence and transmission and highlight how novel technologies such as long read DNA and RNA sequencing are currently being adopted and applied to understand unresolved questions.

Parasitology is becoming more interdisciplinary and several articles in the collection explore the application of novel biophysical approaches. In a review article by Geoghegan et al., the authors discuss proximity labelling, in which a promiscuous biotin ligase is genetically fused to a protein of interest that can then be activated to “tag” nearby molecules. Furthermore, the authors comment on how this cutting-edge method is being implemented to study the intracellular organisation of kinetoplastids, a group of eukaryotic parasitic protozoa of medical and veterinary importance, and emerging trends in the proteomics field. On a separate review, Introini et al. explore the biomechanics of host-pathogen interactions, with an emphasis on how *Plasmodium* parasites manipulate the biophysical properties of red blood cells to their advantage, but concomitantly impacting disease pathogenesis. The authors discuss methods currently being employed to reveal new aspects of *Plasmodium falciparum* invasion of red blood cells and cytoadhesion to host vasculature, including promising techniques from the fields of bioengineering, immunomechanics, and soft matter physics. These methods could be easily transferred in the future to study other stages of falciparum malaria, or to better understand how other parasites interact with their host.

Although technological advances in microscopy have often focused on the improvement of the resolution at the microscopic and nanoscopic scale, light sheet microscopy has recently emerged with the goal to improve temporal and spatial resolution of samples and tissues imaged at a larger scale. In an original research article, Battistella et al. describes how a recently developed light-sheet illuminator coupled to mesoscale imaging has been implemented to resolve neuroimmune interactions in the murine brain during experimental trypanosomiasis. By using whole cleared mouse brain specimens, the authors were able to spatially resolve the localisation of brain cells activated upon infection and proposed that the circumventricular organs are active sites for anti-parasitic responses in this model of infection. This article is the first report highlighting the potential of novel optical approaches such as mesoscale imaging and organ clearing to study immunity against protozoan parasites.

Another big challenge in parasitology is the development of new *in vitro* culture methods. Current culture technologies are far away from reproducing the complex physiology of the host, and this often prevents the successful culture of multiple parasite species. The last section of this collection encompasses a series of articles exploring the use of organoids and organs-of-chips technologies applied to parasitology. In an original article by Faber et al., the authors report for the first time the development of a bovine gastric epithelial organoid from abomasal gastric tissue. The authors show that these newly formed organoids share many structural and transcriptional features from the tissues they were derived from and demonstrated that these organoids can be actively infected by and responded to the pathogenic gastrointestinal (GI) parasite *Ostertagia ostertagi*. White et al. further provide a critical review of the used or intestinal organoids to study infection by GI parasites, outlining applications and important considerations such as developmental stages and culture methods to closely mirror the molecular events taking place *in vivo*. Lastly, Herraiz et al. discuss how humanised mouse models and *in vitro* organ-on-a-chip technologies can be implemented to study *Plasmodium vivax* infection, whose culture still remains unavailable. These methodologies can be use in the future to explore parasite cryptic niches such as the liver, bone marrow or spleen. They also discuss current challenges in this field of research, and how these novel *in vitro* technologies can close the knowledge gap for parasite stages directly contributing to pathogenesis in humans.

Together, this Research Topic provides novel and thought-provoking discussions along with insights in the field of parasitology and serves as a reminder that new discoveries emerge when different disciplines come together to tackle complex biological problems.

## Author contributions

All the authors wrote the manuscript, contributed to manuscript revision, and approved the submitted version.

## Funding

Sir Henry Wellcome postdoctoral fellowship (221640/Z/20/Z to J.F.Q.), Wellcome Trust ISSF Catalyst grant awarded to J.F.Q. (204820/Z/16/Z to JFQ), NIH R00 pathway to independence award (R00AI137218 to DH).

## Conflict of interest

The authors declare that the research was conducted in the absence of any commercial or financial relationships that could be construed as a potential conflict of interest.

## Publisher’s note

All claims expressed in this article are solely those of the authors and do not necessarily represent those of their affiliated organizations, or those of the publisher, the editors and the reviewers. Any product that may be evaluated in this article, or claim that may be made by its manufacturer, is not guaranteed or endorsed by the publisher.

